# The 2016 classification criteria for primary Sjogren’s syndrome: what’s new?

**DOI:** 10.1186/s12916-017-0837-1

**Published:** 2017-03-31

**Authors:** Franco Franceschini, Ilaria Cavazzana, Laura Andreoli, Angela Tincani

**Affiliations:** 1 Rheumatology and Clinical Immunology Unit, ASST Spedali Civili, Brescia, Italy; 2 Clinical and Experimental Science Department, University of Brescia, Brescia, Italy

**Keywords:** Sjogren’s syndrome, Classification criteria, ACR, EULAR, Anti-Ro/SSA, Anti-La/SSB, Ocular staining score, Extra-glandular features

## Abstract

New 2016 ACR/EULAR classification criteria for primary Sjogren’s syndrome (SS) have been developed and endorsed by the ACR. The newly proposed criteria include simple-to-perform items.

Two important points of the new criteria should be considered. Firstly, they indicate that either salivary gland biopsy or anti-Ro must be positive in order to corroborate the inflammatory and autoimmune nature of the disease. Secondly, the criteria recognize the systemic nature of SS, namely that patients without salivary or ocular glandular symptoms, but with extraglandular manifestations and B cell activation markers were also included in the SS classification. Additionally, the new criteria modified some technical points. The ocular staining score threshold was increased to 5 due to the higher specificity. The immunological profile includes only anti-Ro antibodies, while positivity for antinuclear antibodies and rheumatoid factor or isolated anti-La was excluded due to a lack of specificity.

The 2016 ACR/EULAR criteria are suitable for early identification of SS, providing patients with the opportunity of enrollment in clinical trials for new specific treatment. Although validation has been successful, the real life application of these criteria will test their performance.

## Background

Sjögren’s syndrome (SS) is a systemic disease with inflammatory chronic involvement of exocrine glands, mainly ocular and salivary glands. Other organs may be involved in more than 30% of cases and, occasionally, extra-glandular manifestations can occur early during the course of the disease [1, 2]. Although disease pathogenesis has not yet been fully elucidated, substantial data has demonstrated that SS is an autoimmune disease, with autoantibodies to Ro and La acting as its immunological markers [3].

Since 1965, a number of classification criteria sets for SS have been proposed by single experts or groups of multidisciplinary specialists [4–10]. Over the past 15 years, two sets of criteria have been published. The first was proposed by the American-European Consensus Group (AECG) in 2002, and has been extensively used for research and clinical purposes [10]. The second, more recent set was published by the Sjögren’s International Collaborative Clinical Alliance (SICCA), funded by the National Institutes of Health and provisionally endorsed by the American College of Rheumatology (ACR), with the aim to classify patients for enrollment in clinical trials [11]. A comparison between AECG and ACR criteria, performed in 2014, found an excellent concordance rate of 0.81, but also clearly showed that some items, especially for ocular involvement, needed further revision [12]. Thus, the existence of two scientifically validated, similarly performing set of criteria, despite their major differences, led the scientific community to recognize the need for an international consensus on classification criteria. Under the umbrella of the European League against Rheumatism (EULAR) and ACR, investigators from the AECG and SICCA groups collaborated to develop the 2016 ACR/EULAR classification criteria for primary SS [13].

## Discussion

### Was there really a need for a new set of criteria for SS?

AECG criteria comprise a number of positive features, including their simple application and the stepwise approach to patient classification in a rheumatologist’s office without the need for invasive tests provided that ocular and salivary subjective items along with Schirmer’s test and anti-Ro are satisfied. Similarly, SICCA criteria are also simple, but rely only on objective tests, two of which (ocular staining score and lip biopsy) are invasive and cannot be performed in an outpatient setting. Nevertheless, they do not consider radiological and scintigraphic gland examination, which are presently rarely used for diagnostic purposes. A limitation in both sets of criteria was the lack of early detection of SS. However, there was sufficient data to justify the amalgamation of the two sets of criteria into a single consensus formulation. Therefore, the 2016 ACR/EULAR criteria were formulated to combine features from both the SICCA and AECG criteria.

The methodology used in the development of the 2016 ACR/EULAR criteria for SS mirrored that used for the successful development and validation of the ACR/EULAR criteria for rheumatoid arthritis [14] and systemic sclerosis [15], providing a simple scoring system applicable in daily routine clinical practice. Unfortunately, in the absence of an objective “gold standard” to define the disease, the clinician-expert opinion and consensus still represent the best instruments to build a criteria set, as indeed expressed by the authors [13].

### Innovative features of the new classification criteria

The newly proposed criteria include only objective and rather simple-to-perform items. In addition, they are based on a weighted sum of items easily applied in real-life clinical settings.

Objective ocular and oral symptoms have been excluded; nevertheless, they continue to be considered important in evoking the clinical suspicion of SS and guiding the performance of clinical tests.

In this scenario, two other important findings were considered. Primarily, as in AECG criteria, either one of salivary gland biopsy and anti-Ro must be positive in order to emphasize the inflammatory and autoimmune nature of the disease. Secondly, the systemic nature of SS was recognized, allowing the classification of SS for patients even without salivary and ocular symptoms, but with extraglandular manifestations and B cell activation markers. It is well known that, not infrequently, SS is diagnosed after the onset of interstitial nephritis [16] or neurological involvement [17]. Furthermore, these classification criteria could lead to earlier recognition of a patient having SS, because systemic symptoms and/or B cell activation markers are frequently occurring in young patients, with no or scanty symptoms of sicca. Given the purpose of the 2016 criteria of also identifying patients for clinical trials, these young SS patients could particularly benefit from the new therapeutic options.

The new criteria also modified certain technical issues. The ocular staining score threshold was increased to 5 due to the higher specificity [12], compared to the previous score of 3 [11]. The immunological profile includes only anti-Ro antibodies, while positivity for antinuclear antibodies and rheumatoid factor or for isolated anti-La was excluded. Antinuclear antibodies and rheumatoid factor positivity had been included as SS criteria in many previous classification sets [7, 8, 11], but were considered too unspecific to be confirmed in the new criteria. The exclusion of isolated anti-La was based on reliable data showing that anti-Ro antibodies are usually detected either solely or concomitantly with anti-La, whereas anti-La antibodies only seldom exist in isolation [18]. The use of highly sensitive multiparametric assays frequently leads to the detection of isolated anti-La antibodies, with consequent difficulties in results interpretation. The Ro/La system is considered a heterogeneous antigenic complex, constituted by small RNA particles and three proteins (namely Ro52, Ro60 and La), bound together, with peculiar antigenic properties [3]. The various assays do not perform equally in anti-Ro and anti-La detection, depending on the antigenic source [3]. However, there is no mention in any of the classification criteria for SS of the laboratory methods for autoantibody determination; nevertheless, particular care should be considered for anti-Ro detection since the majority of anti-Ro antibodies target conformational epitopes. All assays, whether automated or manual, should include conformational Ro antigens to obtain accurate results. Anti-Ro antibodies represent a crucial item for the diagnosis of SS – the occurrence of both anti-Ro60 and anti-Ro52 antibodies, years prior to diagnosis, has been recently demonstrated to show the highest positive predictive value for future development of SS, especially in younger subjects [19].

## Future directions and conclusions

The new criteria are open for future modifications such as the introduction of anti-Ro profile or parotid ultrasonography. However, the hypothesis to improve serological determination to distinguish between monospecific antibody assays of Ro60 and Ro52 antibodies does not seem particularly useful considering that isolated anti-Ro52 antibodies are frequently found in different autoimmune diseases and are not specific to SS [20].

With the omission of scialography and salivary scintigraphy, which were included in AECG criteria [8, 10], the oral component of the disease is now assessed by labial salivary gland biopsy and whole unstimulated salivary flow. No other radiological investigation was considered useful. Nevertheless, if precise guidelines allowing the standardization and eradication of inter-observer variability of salivary gland ultrasonography become available, then this technique could be considered for inclusion in the criteria. Indeed, the high accessibility, low cost, and non-invasiveness make ultrasonography an important tool for salivary gland assessment in patients with SS [21].

The strength of the 2016 ACR/EULAR criteria lies in their appropriateness for early identification of SS, providing patients with the opportunity to be enrolled in clinical trials for new disease-modifying drugs for SS. Although the exercise of validation has been successful, the real life application of these criteria will test their performance.

## Abbreviations

ACR: American college of rheumatology
ECG: American-european consensus group
EULAR: European league against rheumatism
SICCA: Sjögren’s international collaborative clinical alliance
SS: Sjögren’s syndrome
